# Two-phase importance sampling for inference about transmission trees

**DOI:** 10.1098/rspb.2014.1324

**Published:** 2014-11-07

**Authors:** Elina Numminen, Claire Chewapreecha, Jukka Sirén, Claudia Turner, Paul Turner, Stephen D. Bentley, Jukka Corander

**Affiliations:** 1 Department of Mathematics and Statistics, University of Helsinki, PO Box 68, 00014 Helsinki, Finland; 2 The Wellcome Trust Sanger Institute, Wellcome Trust Genome Campus, Hinxton CB10 1SA, UK; 3 Department of Biosciences, University of Helsinki, PO Box 56, 00014 Helsinki, Finland; 4 Shoklo Malaria Research Unit, Mahidol-Oxford Tropical Medicine Research Unit, Faculty of Tropical Medicine, Mahidol University, Mae Sod, Thailand; 5 Centre for Tropical Medicine, Nuffield Department of Medicine, University of Oxford, Oxford, UK; 6 Department of Medicine, University of Cambridge, Addenbrookes Hospital, Cambridge CB2 0QQ, UK

**Keywords:** transmission tree, molecular epidemiology, *Streptococcus pneumonia*

## Abstract

There has been growing interest in the statistics community to develop methods for inferring transmission pathways of infectious pathogens from molecular sequence data. For many datasets, the computational challenge lies in the huge dimension of the missing data. Here, we introduce an importance sampling scheme in which the transmission trees and phylogenies of pathogens are both sampled from reasonable importance distributions, alleviating the inference. Using this approach, arbitrary models of transmission could be considered, contrary to many earlier proposed methods. We illustrate the scheme by analysing transmissions of *Streptococcus pneumoniae* from household to household within a refugee camp, using data in which only a fraction of hosts is observed, but which is still rich enough to unravel the within-household transmission dynamics and pairs of households between whom transmission is plausible. We observe that while probability of direct transmission is low even for the most prominent cases of transmission, still those pairs of households are geographically much closer to each other than expected under random proximity.

## 1. Introduction

Infectious pathogens lead their lives at the brink of a contradiction: the infection itself typically harms the host, yet the pathogen is somewhat reliant on the well-being of the host, at least on its capability to transmit the infection further. When studying pathogen populations from the perspective of transmission, one is faced with a diversity of different strategies for the pathogen to persist through transmission: vector-borne, air-borne, aggressive, asymptomatic, seasonal and endemic, to name a few. Owing to the complicated modes of transmission, and the pathogen and host heterogeneity, there can exist large variation in the number of secondary infections an infected individual produces [1]. Understanding this heterogeneity yields apprehension about the ecological and evolutionary dynamics and constraints of the pathogen populations.

Molecular sequence data from pathogen populations have recently become increasingly available, bringing new opportunities for the statistical analysis of the processes of transmission. Sequence data allow for fitting transmission models to time-series data [2], or estimating the effective reproduction ratios of the pathogen population in the past [3]. Appropriate analysis can disentangle the time of the peak of the epidemic [4], identify differences between transmission clusters [5] or quantify the underlying contact patterns of the hosts [6].

One particular type of analysis of transmissions is to study the actual transmission trees (i.e. the progression of the transmissions from host to host; see §2b). Recently, Ypma *et al*. [7] and Morelli *et al*. [8] have proposed statistical inference frameworks for transmission trees, based on joint analysis of data on symptom times and genotypic data of the pathogenic isolates. These methods have been further extended by taking the within-host evolution of the pathogen into account [9], and developing tools for analysis when a big fraction of hosts was unobserved that are able to take the depletion of susceptible hosts into account in a mechanistic way [4,5]. Generally, the number of units contributing to an epidemic is large, and many of the hosts that contributed to the spread remain unobserved. For instance, Blum & Tran [10] predict the total size of HIV epidemic in Cuba to be 42 000 by the year 2015 and only 40% of the cases to be detected by the screening system. An explicit data augmentation scheme [11] provides a solution for the statistical inference of transmission links in the case of missing data. However, constructing an effective proposal density for the unobserved data remains a challenge, and if not done properly may lead to biased results, as observed for instance in a study by Aandahly *et al.* [12].

Many of the earlier proposed methods for transmission trees are based on MCMC methods and thus require efficient proposal densities for the unobserved data. In these situations, the quality of the approximations might trade off with the complexity of the considered model. As an alternative, we present a framework inspired by importance sampling that allows for considering arbitrarily complex transmission models, the only requirement being that transmission trees are easy to sample under it. This approach is particularly suitable for situations in which some features of the transmission process are known *a priori* and the aim is to use that knowledge for the inferences. As an example of our framework, we study transmissions of the mostly asymptomatic bacterium *Streptococcus pneumoniae* from one household to another in a refugee camp. A dynamical model was used as an importance distribution for transmission trees, when conditioning on the data on genotypes of the isolates. For the refugee camp data, we predict that the probability of direct transmission highly depends on the assumptions on the observation process, but is often less than 0.3 even for genetically and temporally closely related pairs of isolates.

## 2. Material and methods

### (a) Data

#### (i) Study design

There exist over 90 serotypic strains of *S. pneumoniae*, a bacterium that colonizes the human nasopharynx. While the colonization is often asymptomatic, children and elderly people especially are susceptible to pneumococcal diseases, such as pneumonia or meningitis. The data we analyse here come from a study on pneumococcal carriage among infants and their mothers living in a long-term refugee camp, Mae La, situated in northwest Thailand. For these data, 250 mother–infant pairs were recruited to be sampled regarding their pneumococcal colonization status each month over 2 years. Furthermore, the colonizing strains of an additional 750 infants were screened during the presence of clinically diagnosed pneumonia. In total, 6747 pneumococci were isolated from all the 11 829 swabs taken and the whole genomes of 3085 isolates were sequenced. For full details and references to the original data, see [13]; for exploratory analysis of it, see [14].

#### (ii) Parsimonious phylogenetic trees

Using the method described by Croucher *et al.* [15] and the sequence information available, we generated parsimonious maximum-likelihood trees in which the branch lengths can be interpreted as accumulations of new single nucleotide polymorphisms (SNPs). The trees were parsimonious in the sense that they required least mutations to be assumed. In addition, only those SNPs that have a negligible probability of being due to recombination were considered in the construction. For details on removing recombination, see [13].

#### (iii) Longitudinal observations from households

The data can be rearranged into observations from different households. Those mother–infant pairs that were sampled monthly yield longitudinal observations on the colonization dynamics within the whole household they live in. In §2 in the electronic supplementary material, we show a few examples of such longitudinal observations from households. The observations from households are partial, as not all the household members were screened, and the exact household sizes are unknown. However, there are general demographic data available about the camp in [16], which can be used for modelling purposes.

#### (iv) Plausible transmission pairs and transmission clusters

For our study, we assume that if the same serotype was observed to colonize mother and infant in consecutive sampling times, there was a direct transmission between them. Using these training examples, we construct a classifier for pairs of observations *in different households* that classifies the transmission to be plausible between the two households. Using this classifier and parsimony tree of the isolates, we cluster data into smaller sets of data, so that the pairs of isolates that indicate a plausible transmission between the two households are clustered together. In addition, the isolates within clusters can be assumed to have emerged from small separate epidemics within the camp, and thus can also be analysed separately. We describe the details of the classifier and the data partitioning in the electronic supplementary material.

### (b) Importance sampling of transmission trees

We use the term *transmission tree* to refer to a graph that is a complete description of an epidemic. It contains information on the exact time span and the source of infection for each host, apart from the first infected individual. For a mathematical definition of a transmission tree, see [17]. We start from the assumption that it is possible to construct a sampling distribution for transmission trees that captures *a priori* known relevant features of the transmission process. We denote this distribution with *Γ*(*·*), and the trees sampled from it by *Z* (table 1 summarizes all the notation used in this article).

**Table 1.** The most important notations and concepts used in the article.

*Z*	transmission tree
*Γ*(*·*)	proposal distribution for sampling transmission trees, i.e. *Z* ∼ *Γ*(*·*)
***T***	the genealogy of the considered isolates in a transmission cluster; here ***T*** is a matrix that describes the time to most recent common ancestor for all the pairs of considered isolates
***T****_Z_*	a matrix describing for two different infectees the time of coalescence *along the transmission tree*
*Λ*(*·*)	proposal distribution for sampling the genealogy of the isolates (i.e. ***T*** ∼ *Λ*(*·*)) we use the posterior distribution of ***T*** as *Λ*(*·*)
*S_Z_*(*k*)	a set all the different sequences of *k* branches of tree *Z*, each extant during the follow-up
***x***	a particular combination of branches, i.e. ***x** ∈ S_Z_*(*k*)
*Ψ*(*·*)	posterior predictive distribution for the time span of infectiousness for a household; in our application this corresponds to the distribution for the branch lengths for transmission trees *Z*
*r*	the rate at which infectious households infect each other

Consider data collected in time interval [*t*_s_, *t*_e_] according to a known observation process. Assume the data consist of *k* isolates, with genotypes ***g***, observed at times ***t*** in different hosts. Assume also that it is possible to sample from the posterior distribution of the genealogies of the observed isolates conditional on ***g*** and ***t***. Denote this distribution with *Λ*(*·*), and realization from it by ***T***.

The sampling scheme we propose is importance sampling using two importance distributions, *Λ*(·) and *Γ*(·). First, ***T*** is sampled from *Λ*(·). This defines the time origin for *Z* that is sampled subsequently from *Γ*(·). *Z* is initiated at the time to most recent common ancestor (TMRCA) of the isolates in *T*. Finally, the importance weight of the proposed *Z* is calculated by evaluating the probability of temporal observations and the genotypes.

Our main focus is on posterior inference about transmission trees given data and a model for both sequence evolution and transmission. A direct inference approach based on standard Bayesian MCMC computation is very difficult owing to model complexity and the large underlying space of unobserved events that would need to be explicitly simulated. Vague prior distributions do further escalate this challenge. Therefore, we develop an alternative approach where a sensible importance distribution is used to sample both transmission trees and genealogies to reach an approximation of the target distribution. The motivation for taking this route to posterior approximation is that both genealogies and transmission trees are easy to sample and the data already define to a considerable degree what characteristics they must have.

#### (i) The importance weight of *Z*

For evaluating the likelihood of observations for a proposed *Z*, we consider all different vectors of length *k* in which the elements correspond to different branches of *Z* that were extant during the follow-up [*t*_s_, *t*_e_]. We denote this set by *S_Z_*(*k*), and the elements of this set with ***x***. Furthermore, we denote with {*k* = *O*(*Z*)} the event that a total of *k* branches were observed from tree *Z* during the follow-up. In addition, {***x***, ***t***|*Z*)} denotes the event in which exactly the branches ***x*** were observed exactly at times ***t*** from *Z*. Finally, with {***g***|***x***, ***t***, *Z*}, we denote the event that the isolates collected at times ***t*** from branches ***x*** of the tree *Z* would have genotypes as in ***g***.

Because *Γ*(·) can be regarded as the prior distribution for *Z*, the target of our inference is the posterior distribution *P*(*Z|**t***, ***g***) = *c* × *P*(***t***, ***g****|Z*)*Γ*(*Z*), where *c* is a constant. The importance weight, *w*(*Z*), of proposed tree *Z* is the ratio of the probability of *Z* under target distribution to the probability of *Z* under the proposal distribution. Because *Γ*(·) is also the proposal distribution, and *c* does not depend on *Z*, the importance weight can be defined as the likelihood *P*(***t***, ***g|****Z*). The likelihood factorizes in terms of *S_Z_*(*k*)2.1
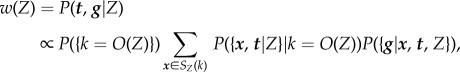
where *P*({***x***, ***t****|Z*}*|k* = *O*(*Z*)) is the conditional probability of observing branches ***x*** at times ***t*** from *Z*, conditional that *k* branches were observed from *Z* during the follow-up. If (2.1) was intractable, the observation process can be simulated several times on *Z*, and the resulting ‘pseudo-observations’ can be compared with actual data, as done in the methods of approximate Bayesian computation [18]. Conditional on a given tree *Z*, sets of branches have specific weights, given by2.2
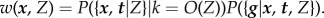


#### (ii) The sampling scheme

The proposed sampling scheme is the following:

(1) Sample a genealogy ***T*** for the isolates from *Λ*(·).
(2) Sample a transmission tree *Z* from *Γ*(·) initiated at the earliest coalescence time in ***T***. Information on branches born after the end of the follow-up *t*_e_ can be disregarded.
(3) Allocate importance weights *w*(***x***, *Z*) to sets of branches for all ***x****∈S_Z_*(*k*) as defined in equation (2.2).
(4) Allocate an importance weight for the sampled tree2.3
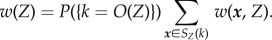

**Figure 1.**
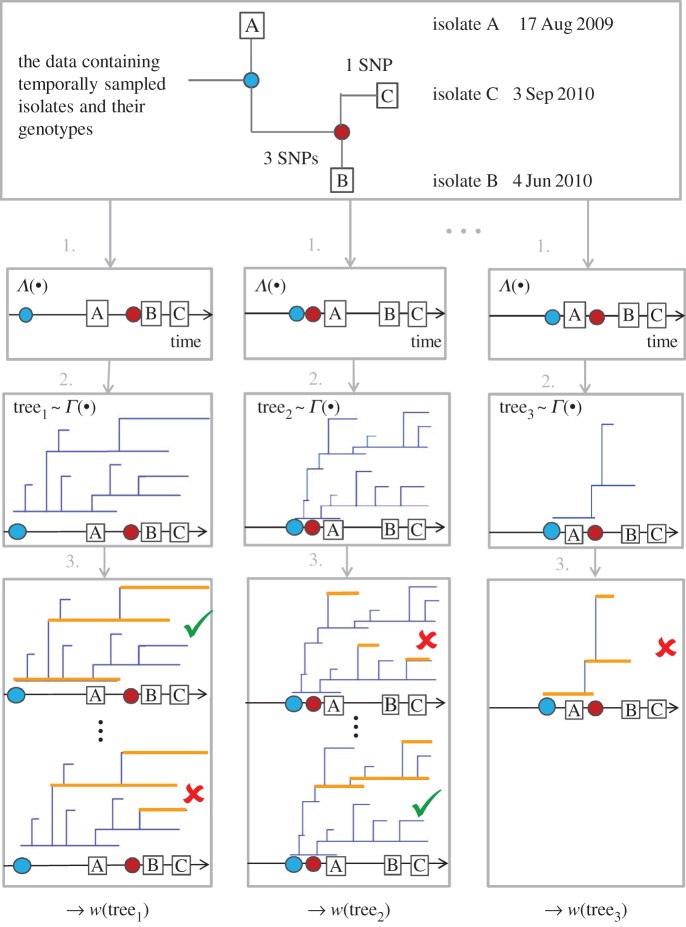
Our framework begins by (1) sampling the unknown coalescence times of isolates from their posterior *Λ*(*·*), and mapping them to the same time axis with the observation times of isolates. In (2), transmission tree is proposed from *Γ*(*·*) and initiated at TMRCA of all the isolates that were sampled previously. For calculating the importance weight of transmission tree, we evaluate the likelihood that under the observation model the resulting observations are consistent with both the genealogy and observation times (3). By repeating steps (1), (2) and (3), a weighted sample of transmission trees is obtained for isolates A, B and C. In the illustrated transmission trees, horizontal lines denote infectious periods within one host, and vertical lines denote new infections from that host. In (3), we have illustrated potential outcomes of the observation process by marking the observed infections in orange. Some of such observations would be incompatible with information in step (1) and lead to a likelihood of zero of being observed. For instance, the ‘pseudo-observations’ in the middle panel at top would yield zero contribution to the importance weight, because none of the orange lines were alive at observation time B. Similarly, the bottom example in the left panel is inconsistent with observations, because it is impossible allocate the labels A, B and C to the orange branches so that each branch would be alive at the corresponding observation time and the lineages of isolates B and C could coalesce at the required time (denoted by the red dot). Finally, the rightmost transmission tree will be assigned zero weight, because there are no extant infections at the observation time C.

We illustrate the proposed sampling scheme in figure 1. Above steps 1–4 are repeated until sufficiently many transmission trees with considerable importance weights are obtained. After the sampling, the obtained importance weights, *w*(*Z*), are normalized, and the transmission trees are resampled according to the weights. Subsequently, for each resampled transmission tree, a vector *x∈S_Z_*(*k*) is sampled with a probability proportional to *w*(***x***,*Z*). Then, the distribution of the number of transmission links between the sampled ***x*** in the corresponding trees corresponds to the posterior distribution of transmission links between the actual hosts in the data.

#### (iii) Constructing *Γ* (·) for the refugee camp data

For the Mae La data, we were interested in studying transmissions from household to another. To obtain a sampling distribution *Γ*(·) for household-to-household transmission trees, we first fitted a transmission model for *within-household transmission dynamics*, similar to [18], and to longitudinal serotypic observations from households. The model assumes that household members face a constant infection pressure from the community, but transmissions between the household members occur as in a stochastic multi-strain susceptible–infectious–susceptible (SIS) model, with competition between the strains. We allowed the adults to have different susceptibility and rate of clearance than the infants, which corresponds to the immunity that is gained with age. The description of the model and the fitting procedure together with the results are provided in the electronic supplementary material. The posterior distribution of the parameters of the within-household transmission process induces a posterior predictive distribution of the time period an infection circulates within a household, denoted with *Ψ*(·).

We assume that each household is equally infectious as long as at least one household member is infectious. At the metapopulation level, we assume the population of households is well mixed and pneumococcal population is at its endemic equilibrium, which is supported by the exploratory analysis of the data (see electronic supplementary material) [14]. Therefore, each infected household is expected to infect on average one other household. This assumption, together with the distribution for the infectious periods, allows us to solve the rate *r* at which a household infects other households from the equation2.4
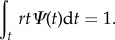
Information on *r* together with the distribution *Ψ*(·) yields *Γ*(·), from which it is easy to sample as shown in the electronic supplementary material. We also show in the electronic supplementary material, figure S4 that there is not significant seasonality in colonization, which validates our assumption on constant *r*.

#### (iv) The alternative observation models

To assess the importance weight in steps 3 and 4 of the sampling, one needs to define an observation model for data collection, defining the probabilities for events {*k* = *O*(*Z*)} and {***x***, ***t***|*Z*)}. This observation model should mimic the process under which data were collected, and incorporate features such as whether infections (i.e. branches) were observed independently of each other, whether there were some intensive sampling periods, and whether infections are more likely to be observed within a certain phase of infection.

In addition, a model for genotypes within transmission trees that defines probabilities of events {***g***|***x***,***t***,*Z*} is needed. In our application, we set the time origin by sampling a genealogy ***T*** from *Λ*(·) before proposing *Z* from *Γ*(·). In addition, instead of considering *P*({***g***|***x***, ***t***, *Z*}) in the calculation of likelihood in (2.1), we evaluate P({***T***|***x***, ***t***, *Z*}) for the currently sampled ***T***. To see that this is appropriate, let *P*(***g***|***T***) denote the likelihood of observed genotypes given the genealogy ***T*** of the isolates. Then, assuming that ***g*** is conditionally independent of other information given ***T***, we may write ∑***_T_****P*(***g****|**T***)*P*(***T****|**t***, ***x***, *Z*) as the likelihood of genotypes given a transmission tree *Z* and the observed ***t***, ***x***. Assuming a constant prior on the genealogies and noting that *P*(***g****|**T***) is proportional to the posterior *P*(***T****|**g***), we may effectively approximate the likelihood calculation by simulating genealogies ***T*** from the posterior *P*(***T****|**g***) and evaluating *P*(***T****|**t***, ***x***, *Z*) for them. Alternatively, one could also consider the probabilities of events {***g****|**x***, ***t***, *Z}* directly.

In our application, for ***T***, we consider only the coalescence times of for all the isolates in the data (i.e. ***T***(*i*,*j*) defines the TMRCA for isolates *i* and *j*). To define *P*(***T****|**x***, ***t***, *Z*), we thus specify a model that defines for every pair *i*, *j* of branches of in the tree *Z* the probability of coalescence at time ***T***(*i*,*j*). We denote with ***T****_Z_* a matrix that is obtained from a transmission tree *Z*, which defines for every two infected individuals the time in the past when their lineages along the transmission tree coalesce to a single host. For instance, if ***T****_Z_*(***x***(*i*), ***x***(*j*)) < ***T***(***x***(*i*), ***x***(*j*)), then *P*({***T***|***x***, ***t***, Z}) = 0. In general, *P*({***T***|***x***, ***t***, Z}) would depend on within-host population sizes of the pathogen and the possible transmission bottlenecks.

We call sets of branches ***x*** of *Z consistent* with data and *T* if for every pair of branches in *i*, *j∈****x*** it holds that ***T****_Z_*(*i*, *j*) > *T_i_*_,*j*_ and if for every observation time in the data ***t***(*i*) the corresponding branch *i* of *Z* is extant at ***t***(*i*). For each set of branches that is not consistent with data and ***T***, it will always hold that *w*(***x***, *Z*) = 0. How the consistent sets of branches are further weighted against each other depends on the further assumptions made on the observation process.

The data considered here were not collected according to a specific process, as the genotypes of isolates were sequenced selectively and also the swabs were collected both routinely and occasionally from diseased children. To study the robustness of our conclusions, we considered the following six alternative models for observations:

— *Model 1*. Infections are observed independently of each other with a probability *p*. The actual time they are observed is uniformly distributed over the time of infectiousness.
— *Model 2.* Infections are observed independently of each other with a probability *p*. The actual time an infection is observed is uniformly distributed over the time of infectiousness, if the infection period was shorter than one month. If the infection period was longer than a month, the time of observation has a truncated gamma distribution centred at the 30 days from the beginning of the infection. Thus, long infection periods are more probably observed at the beginning of the infectious period.
— *Model 3*. Infections are observed independently of each other, but the probability of an infection being observed is proportional to the length of the infectious period. The actual time they are observed is uniformly distributed over the time of infectiousness.
— *Model 4.* Observations are collected as in model 1, but *P*({***T***|***x***,***t,***Z}) is defined so that genotypic lineages, once in the same host, are more likely to coalesce during the infection period of the host than to remain as separate lineages that were both transmitted from the source of infection.
— *Model 5*. In this model, data are collected as in model 3, but the transmission bottlenecks are penalized as in model 4.
— *Model 6.* Data are collected as in model 2, and the transmission bottlenecks are penalized as in model 4.

In the electronic supplementary material, we show how the elements of the importance weights in equation (2.3) can be calculated under each of the six models above.

## 3. Results

To illustrate the framework presented here, we implemented the presented scheme on observations considering the most prevalent serotypes. The data were clustered as described in the electronic supplementary material. This resulted in 53 separate data clusters with a total of 103 pairs of households among whom transmission was considered plausible. Each data cluster was analysed independently of the others using the framework described above. In our application, we assumed the topology of the genealogy to be fixed for each cluster and given by the parsimony tree, and sampled only the coalescence times from the posterior. In the electronic supplementary material, we show how we sample from the posterior distribution of ***T***, conditional on the parsimonious phylogenetic tree. Assuming fixed genealogy might produce overconfident results considering transmission links, when data clusters of more than two isolates are considered. Therefore, the topology could also be considered uncertain, and the genealogies could be sampled using, for instance, standard bootstrap techniques.

### (a) From within-household dynamics to between-household transmission trees *Γ*(·)

The estimated within-household dynamics indicate frequent transmissions between the family members. For a susceptible infant, it is approximately 4.5 times more probable to be infected from any infectious family member per day than from the community outside the household. Because the infections could circulate within the household for very variable amounts of time, the posterior predictive distribution for the lengths of infectious periods *Ψ*(*·*) is predicted to be highly skewed, having a 95% credibility interval (CI) of [1, 345] days, whereas the posterior mean is 62 days. The rate *r* at which infectious households infect each other was estimated to be *r* = 0.016 per day. Combining the inferences about the lengths of the infectious periods for households and the rate of infecting other households, we predict that approximately 53% of the infected households infect no other household, but still 11% of the infected households will infect four or more other households. The mean generation time interval for the household-to-household transmissions is predicted to be 92 days, with 95% CI being [2, 352] days. In figure 3, several examples of transmission trees sampled from *Γ*(*·*) are shown. The statistical properties of *Γ*(*·*) are shown in detail in the electronic supplementary material, figure S3.

### (b) The predicted transmission links

**Figure 2.**
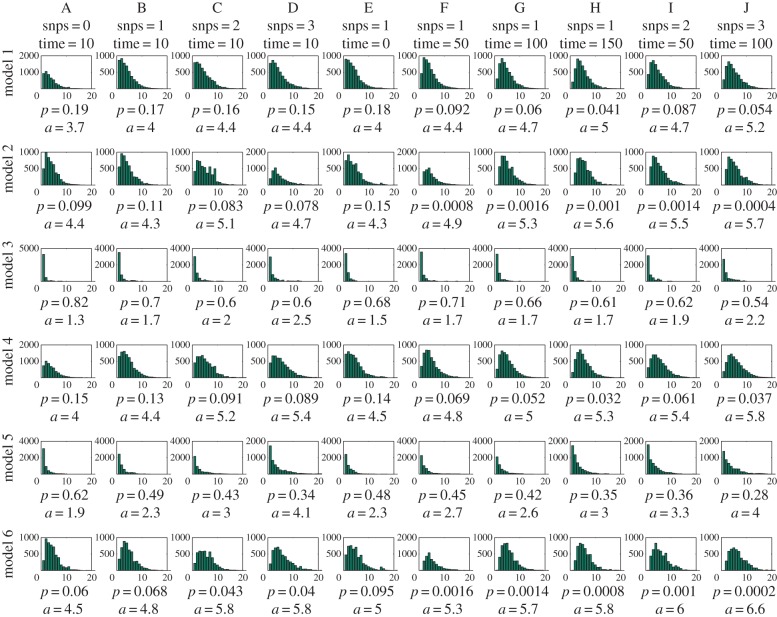
Histogram approximations to the posterior predictive distribution for transmission links. Each column corresponds to a different case of isolates and each row to a different observation model. Below each histogram, we show the estimated probability of direct transmission (*p*) and the posterior mean for transmission links (*a*).

In figure 2, we show for 10 hypothetical pairs of observations and for six different observation models the posterior distribution of the number of transmission links. All these cases were treated as independent of the rest of the data, thus belonging to clusters consisting of two isolates only. As expected, small differences in genomes or between the observation times indicate lesser transmission links between the cases. However, observation model strongly influences the predictions: even for cases A and G, which *a priori* appear likely to be due to direct transmission based on their small genotypic and temporal distance, the probability of direct transmission can vary from 0.2 to 0.8, depending on the assumed observation model. Most direct transmissions are predicted between the cases under model 3, in which the longer infections are more prone to be observed. Under this model, the observations in the data are more likely from relatively long infectious periods and thus also more likely have produced several secondary infections, therefore the number of secondary infections produced by an infection is correlating with the probability of an infection to be observed. On the other hand, inclusion of transmission bottlenecks in the observation model, as in models 4–6, decreases the probability of direct transmission for all the data collection models.

**Figure 3.**
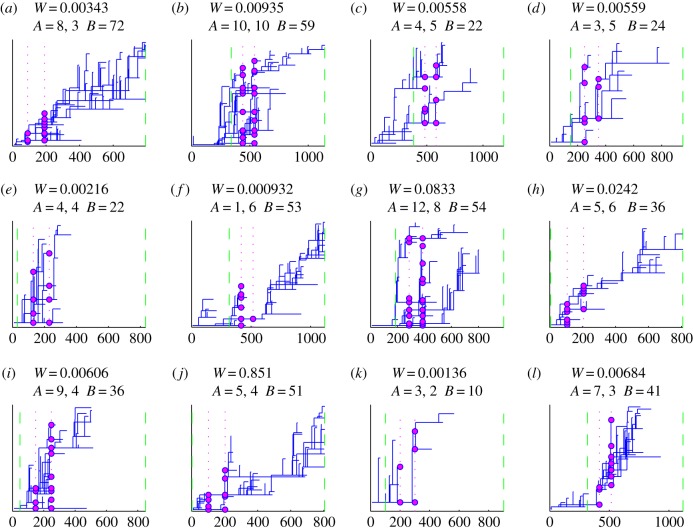
An illustration of the posterior distribution of transmission trees for a pair of isolates having two-SNP distance of one and being observed 100 days apart from each other. The normalized weights of the 12 sampled trees are shown above each tree. We also show the number of branches alive at the first and second observation time (*A*) and the total number of branches born during the follow-up (B). In each panel, time origin was set to the time of the most recent common ancestor. Relative to that, vertical green lines mark the start and the end of the follow-up, and the lines and spheres in magenta show the branches alive at the two observation times.

**Figure 4.**
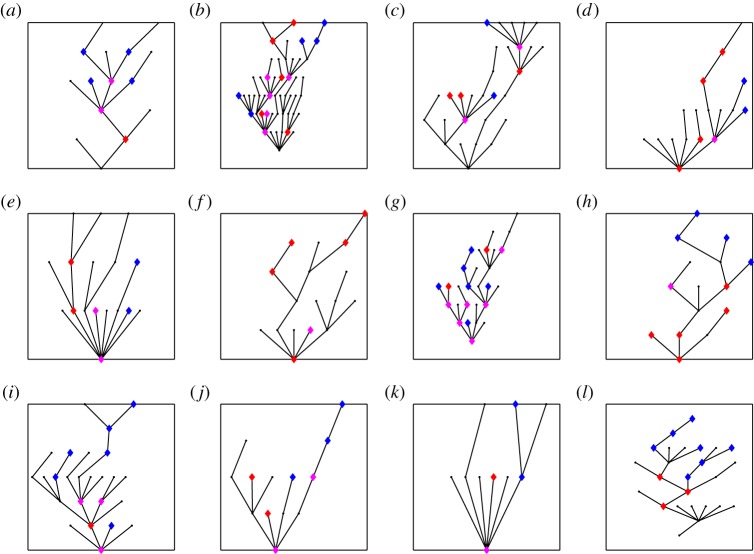
The topology of the subtree tree connecting the branches alive at observation times, for the same trees as shown in figure 3. We show the branches alive at first observation time in red, at both observation times in magenta and at the second observation time in blue.

In figure 3, we show 12 transmission trees sampled from the posterior distribution for case G in previous figure 2, conditional on observation model 4. This is a pair of isolates collected 100 days apart from each other and who have one different SNP. In figure 4, we show for the same trees as in figure 3 the tree topology of the subtree that connects branches alive at the observation times to their MRCA. From these figures 3 and 4, we can assess what sort of trees acquire large importance weights. If a transmission tree has too few branches in total, the likelihood of observing two branches is low, as in case (*k*), whereas if the tree has numerous branches during the follow-up, we would expect to see more than two branches, as in case (*a*). Conditional on a certain total size of the tree, larger weights are obtained when there exist several different pairs of branches, for which the first one is extant at the first observation time (red marks in figure 4) and the second at the second observation time (blue marks in figure 4). The trees getting large weights are thus the ones that have several branches at the times of observations but which go extinct soon after that, but such trees do not have high probability in our branching process model for transmission trees. Still, as the observation model 4 penalizes for transmission bottlenecks, transmission trees such as (*g*) and (*l*) obtain lower weights. This is because a randomly sampled consistent pair of observations might coalesce after several transmissions along the transmission tree, as seen from figure 4.

**Figure 5.**
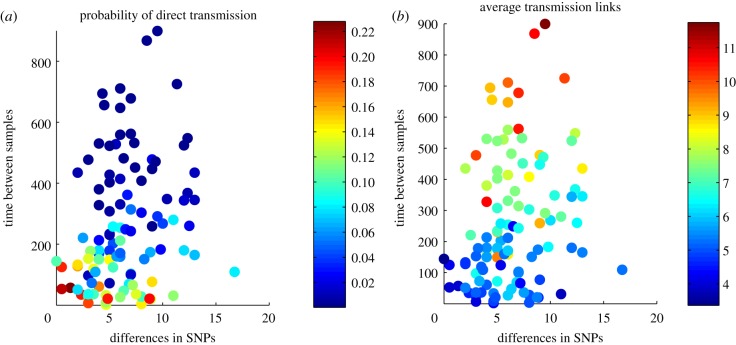
(*a*) The posterior predictive probability of direct transmission and (*b*) the posterior mean for predicted transmission links for the pairs of households in the data among whom transmission was considered plausible.

In figure 5, we summarize the predictions considering the actual Mae La data averaged over the six observation models. In the electronic supplementary material, figures S8 and S9, we show for the different observation models the probabilities of direct transmission and the average number of transmission links predicted by the separate models. For many pairs of isolates with close temporal proximity and almost identical genotypes, the probability of direct transmission is only around 0.2, but the majority of them are linked through fewer than four transmission links. We also see that there exists a lot of variation in predictions between fairly similar pairs of observations. This emphasizes the relevance of the different sources of information, because the pairwise temporal and genotypic distances between pairs of isolates alone are not sufficient for the inferences. Indeed, while the majority of data were clustered into clusters of size 2, in bigger clusters, the information content is different. In addition, the actual times of observations matter. For instance, two observations close to the end of the follow-up could be due to a recent rapidly expanding epidemic, whereas this is not as likely for observations in the beginning of the follow-up, because if the epidemic had a rapid expansion, we would expect to observe more isolates from this epidemic later during the follow-up.

### (c) Interpreting the results

Our results indicate that finding many certain cases of direct transmission is highly unlikely when the carrier population is sparsely sampled, even when the genotype information as such would indicate close relatedness. However, identifying hosts among whom only a few transmission events have occurred can also bring insights about transmissions.

**Figure 6.**
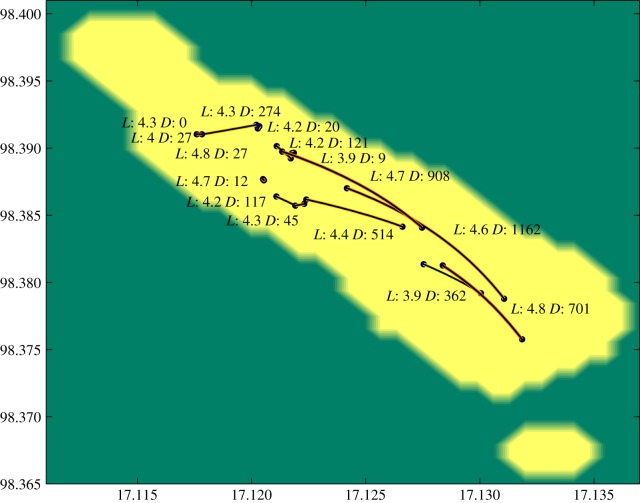
The pairs of households between whom the model average of the predicted number of transmission events was among the 15 closest ranked. The yellow area corresponds to the populated area in the refugee camp, and the points correspond to households, connected by lines presenting possible transmission. Near each line we show the posterior mean for the predicted transmission links (*L*), together with the distance between the corresponding households in metres (*D*). The pairs with highest proximity to each other are the least visible in the figure.

As we had the GPS coordinates of the locations of residence for most of the hosts in the data, we studied the geographical distance of the households who were predicted to be closely related in the transmission tree. We studied the pairs of households for which the posterior mean number of transmission links was lowest. For all six observation models, the closest ranked pairs of households in terms of transmission links also had on average much closer geographical proximity to each other than would be expected by chance. For instance, when considering the posterior mean of links averaged over the six observation models, the 15 closest ranked pairs of households had on average a distance of 286 m from each other, whereas the average geographical distance between all the households in the data is 1022 m. These pairs are shown in figure 6. In the electronic supplementary material, §8, we show in more detail the effect of different observation models, and also the null distribution for the mean distance for 15 random pairs of households, under which the observed 286 m is highly improbable.

While there are differences between the observation models in terms of the predicted transmission links, all the models are consistent with the above spatial observation. This actually constitutes very strong evidence for localized transmissions: if these households are likely to be linked by a few intermediate hosts in the transmission tree and not by a direct transmission, as predicted by our analysis, and they still appear geographically significantly close to each other, the direct transmissions seem even more likely to be localized. Even in a small area like Mae La, with length of few kilometres and width of less than 1 km, transmissions thus occur more often between closer neighbours. It is still unknown whether this is because the neighbours have more direct interaction or, for instance, if they share a water supply.

## 4. Discussion

We have presented a Bayesian approach for analysing transmission events for a partially sampled temporal data of genotypic isolates of infectious pathogens. The approach was based on the insight that some features of the data are informative about the transmission process in general, whereas the other features are mostly informative about the genealogy of the particular isolates collected. Importantly, both types of information are necessary for making inferences about actual transmission trees.

We are aware that there exist various tools for inferring the nature of transmission dynamics directly from the phylogeny [6,2,3,17]. We present an alternative approach based on importance sampling that is not tied to any particular model for transmissions or observations. In many cases, transmission trees are even *a priori* known to have complicated features, such as some particular heterogeneity in the number of secondary infections produced by an infected individual. Such heterogeneity was a predominant feature of the data considered here, because the duration of within-household epidemics had such a large variation. When previous estimates about the transmission dynamics are available, or if some other epidemiological inference methods can be used to unravel them, it is optimal to use such information by introducing a corresponding importance distribution. This is particularly important because recent studies [19,20] suggest identifiability issues if contact structure or other epidemic properties are inferred from phylogeny without any other prior information about the transmission dynamics. Motivated by these findings, we first made inferences about the transmission process without using the sequence data to construct a proposal distribution for transmission trees. Then, each proposed transmission tree was assigned an importance weight by evaluating the overall probability of the observed infections and their genealogy. The key advantage of such an approach is its generality, because it allows for inference of transmission links under any kind of model for transmissions and observations. The characteristics of the transmission model affect the relevant features of the transmission tree, such as the offspring distribution, whereas the observation model allocates a posterior probability for each tree, and consequently has substantial influence on the transmission link probabilities. This was highlighted by our observations on the different predictions under the different observation models. This emphasizes the importance of careful study design and observation model assessment.

To make the analysis computationally tractable, we clustered the observations into smallest possible subsets of data that could be argued to have emerged from a process that was independent of the process that generated the rest of the data. While such clustering can be assumed to exist, it may be more uncertain how best to identify it. Because we observed that the clustering influences the nature of predictions for transmissions, developing statistical methods for this would be a relevant topic for future investigations. As for the computational feasibility, we stress that our approach enjoys a high level of parallelizability, as one simulated transmission tree could be weighted with respect to different data clusters, and different observation models.

The independence assumption for different branches of the tree is not expected to be valid in general. For large populations and endemic pathogens, such as the one analysed here, such an assumption is reasonably justified. For emerging diseases, the assumption of independence could be made to hold by forcing the branching process model to take into account the rate of epidemic growth as a function of time from the beginning of the epidemic. We are aware of the many other simplifying assumptions used in this study concerning the model of evolution, transmission and the observation process. However, our main focus is to present a general conceptual framework that can be modified and extended to suit a wide range of situations, such that transmissions can be considered under interesting models in a feasible manner.

## Supplementary Material

Supplementary materials for the article ‘Two-phase importance sampling for inference about transmission trees’

## Data accessibility

The references to full-genome data are given in reference [13]. The parsimony trees can be found in Dryad (doi:10.5061/dryad.8d2f6).
