# Supplementary material for: Two-phase importance sampling for inference about transmission trees
Source: Proc Biol Sci. 2014 Nov 7;281(1794):20141324. doi: 10.1098/rspb.2014.1324 (PMC4211445; doi:10.1098/rspb.2014.1324)
Supplement: Supplementary materials for the article ‘Two-phase importance sampling for inference about transmission trees’ [file rspb20141324supp1.pdf]

Supplementary materials for the article 'Two-phase importance sampling for inference about transmission trees'

Elina Numminen Claire Chewapreecha Jukka Siren
Claudia Turner Paul Turner Stephen Bentley
Jukka Corander

Contents

1 The stochastic model for within-household transmission dynamics 3

2 Sequential approximate Bayesian computation approach for the within-household dynamics 4
2.1 Sparse longitudinal datasets from households 4
2.2 Sequential sampling from the joint posterior distribution 6

3 Posterior distributions of the parameters of the within-household-transmission model 8
3.1 Prior and posterior distributions 8
3.2 Posterior predictive distributions from the within-household epidemics 9
3.3 Endemicity and possible seasonality of pneumococcal infections . 10

4 Classification of plausible transmission pairs and partitioning of the data 11
4.1 Plausible transmission pairs 11
4.2 Clustering the data 12

5 Alternative models for observations 14
5.1 Model 1 14
5.2 Model 2 15
5.3 Model 3 15
5.4 Model 4 16
5.5 Model 5 16
5.6 Model 6 16

6 Sampling the coalescence times of the genotypic lineages 17
6.1 Construction of phylogenetic trees without recombination 17
6.2 Sampling the unknown coalescence times of the phylogenetic tree 17

7 Geographic spread of infections 18

8 Supplementary figures **23**

List of Figures

1 Longitudinal observations from mother-infant pairs 5
2 Posterior distributions of the parameters of the within-household model 8
3 Predicted properties of within-household transmission and consequently household-to-household transmission trees 9
4 Temporality of pneumococcal colonization 10
5 Training data on transmission pairs 12
6 Illustration of the method of separating transmission clusters . . 13
7 Illustration on how to calculate the number of transmission bottlenecks 17
8 Geographical spread in the light of different observation models . 20
9 Additional information about geographical spread 21
10 The 15 closest transmission pairs when model average is considered 22
11 Posterior probabilities of direct transmission under alternative models 24
12 Posterior probabilities of direct transmission under alternative models 24

List of Tables

1 Posterior and prior distributions of the parameters of the within-household model 9

1 The stochastic model for within-household transmission dynamics

The transmission model for a household is a stochastic version of SIS (susceptible-infectious-susceptible) model similar to the one presented in [1], with the exception that we consider here two types of hosts, adults and infants. In our model we allow adults to have different susceptibility and clearance rates than infants, which is supported by laboratory experiments [2] and also observational studies [3], [4]. We assume that within a household there is constant number of N_a adults and N_i infants. The household members can be infected with N_s different strains from the community, and the strains can co-colonize the hosts. The system-state for a single household is the enumeration of all the colonizing strains for each of the N_i infants and N_a adults, described by an $N_i \times N_s$ matrix $\boldsymbol{I}$ and an $N_a \times N^s$ matrix $\boldsymbol{A}$, for which $\boldsymbol{I}_{ij} = 1$ if infant i is colonized with strain j, and $\boldsymbol{A}_{ij} = 1$ if adult i is colonized with strain j.

We assume that there is a constant force of infection from the community β_c, such that per day, the probability of being infected from the community is β_c for each susceptible infant in the household and $s_t \times \beta_c$ for every susceptible adult, where parameter s_t is used for scaling for the different susceptibility among adults. The infecting strain is assumed to be a random strain from the total serotype distribution across the population, which is also assumed to be stable, so that prevalence of each serotype s is assumed to be P^s. For a susceptible infant, the probability per time unit to become infected with strain s is $P^s \times \beta_c$. We assume that the rate of clearance of an infection is λ for any strain that colonizes an infant and the rate of clearance for adults is $s_c\lambda$ for any colonizing strain. Thus we assume that the rate of clearing an infection with a particular strain does not depend on the number of other strains colonizing the individual. To define the transmission model, we use the following notation, for the probability that strain s is transmitted, given an encounter with a household member:

$$E_s(\boldsymbol{I}, \boldsymbol{A}) = \sum_{i:\boldsymbol{I}_{is}=1} \left(\frac{\boldsymbol{I}_{is}}{N_i + N_a - 1} \frac{1}{\sum_{j=1}^{N^s} \boldsymbol{I}_{ij}} \right) + \sum_{i:\boldsymbol{A}_{is}=1} \left(\frac{\boldsymbol{A}_{is}}{N_i + N_a - 1} \frac{1}{\sum_{j=1}^{N^s} \boldsymbol{A}_{ij}} \right)$$

This corresponds to the probability of sampling strain s in a hierarchical random experiment, where first a random household member is sampled, and then from the strains that colonize this household member, one of them is sampled. The probability for a susceptible infant to get colonized with strain s from a household member is then $\beta_h(E_s(\boldsymbol{I}, \boldsymbol{A}))$ per day, and for a susceptible adult this is $s_t\beta_h(E_s(\boldsymbol{I}, \boldsymbol{A}))$. We assume that the relative rate of a colonized host to be colonized with a new strain, either from household member or from community, is always θ. The model is thus parametrized with six parameters $\boldsymbol{\psi} = (\beta_h, \beta_c, \theta, \lambda, s_t, s_c)$. The dynamics of the model are defined to be the following continuous-time Markov chain, that has the following transition probabilities for a small time increment Δt, for each of the strains s:

$$\begin{cases} Pr(\boldsymbol{I}_{is}(t+\Delta t)=1|\boldsymbol{I}_{is}(t)=0)=\beta_h E_s(\boldsymbol{I},\boldsymbol{A})+\beta_c P^s+o(\Delta t), \\ \qquad \text{if } \sum_k \boldsymbol{I}_{ik}(t)=0. \\ Pr(\boldsymbol{I}_{is}(t+\Delta t)=1|\boldsymbol{I}_{is}(t)=0)=\theta(\beta_h E_s(\boldsymbol{I},\boldsymbol{A})+\beta_c P^s)+o(\Delta t), \\ \qquad \text{if } \sum_k \boldsymbol{I}_{ik}(t)>0. \\ Pr(\boldsymbol{I}_{is}(t+\Delta t)=0|\boldsymbol{I}_{is}(t)=1)=\lambda+o(\Delta t). \\ Pr(\boldsymbol{A}_{is}(t+\Delta t)=1|\boldsymbol{A}_{is}(t)=0)=s_t(\beta_h E_s(\boldsymbol{I},\boldsymbol{A})+\beta_c P^s)+o(\Delta t), \\ \qquad \text{if } \sum_k \boldsymbol{A}_{ik}(t)=0. \\ Pr(\boldsymbol{A}_{is}(t+\Delta t)=1|\boldsymbol{A}_{is}(t)=0)=s_t\theta(\beta_h E_s(\boldsymbol{I},\boldsymbol{A})+\beta_c P^s)+o(\Delta t), \\ \qquad \text{if } \sum_k \boldsymbol{A}_{ik}(t)>0. \\ Pr(\boldsymbol{A}_{is}(t+\Delta t)=0|\boldsymbol{A}_{is}(t)=1)=s_c\lambda+o(\Delta t). \end{cases}$$

Such model assumes ecological neutrality in terms of serotypes, since all serotypic strains are assumed to have the same rates when it comes to colonization and clearance. Only difference between the strains in the model is how prevalent they are in the community, specified by P^s. For P^s, we used the empirical distribution of the strains in all the collected samples in the data. An ecologically neutral model has previously been observed to describe well the patterns of co-occurence of different strains, as discussed in [1].

2 Sequential approximate Bayesian computation approach for the within-household dynamics

2.1 Sparse longitudinal datasets from households

For the inference of the parameters of the transmission model for within-household transmissions, we partition the whole data into longitudinal observations from different households. Then, from each household h we have N_h observations at observation times $\boldsymbol{t}_h$:

$$\{\boldsymbol{I}_h(i), \boldsymbol{A}_h(i), \boldsymbol{t}_h(i), i=1,...,N_h\}, \tag{1}$$

where $\boldsymbol{I}_h(i)$ is the colonization status of the infant at the observation time $\boldsymbol{t}_h(i)$ and $\boldsymbol{A}_h(i)$ is the corresponding status for the mother. With colonization status we mean the enumeration of the colonizing strains, if there are any.

We consider data sets of the type (1) from different households as independent observations, and fit the transmission model to each of these separately. However, we do not consider households from which either mother or infant was observed less than 6 times. This results in 149 within-household data sets to be analysed from the samples collected.

In Figure 2.1 we show a few examples of the longitudinal observations from households. From the examples we observe that typically many different strains

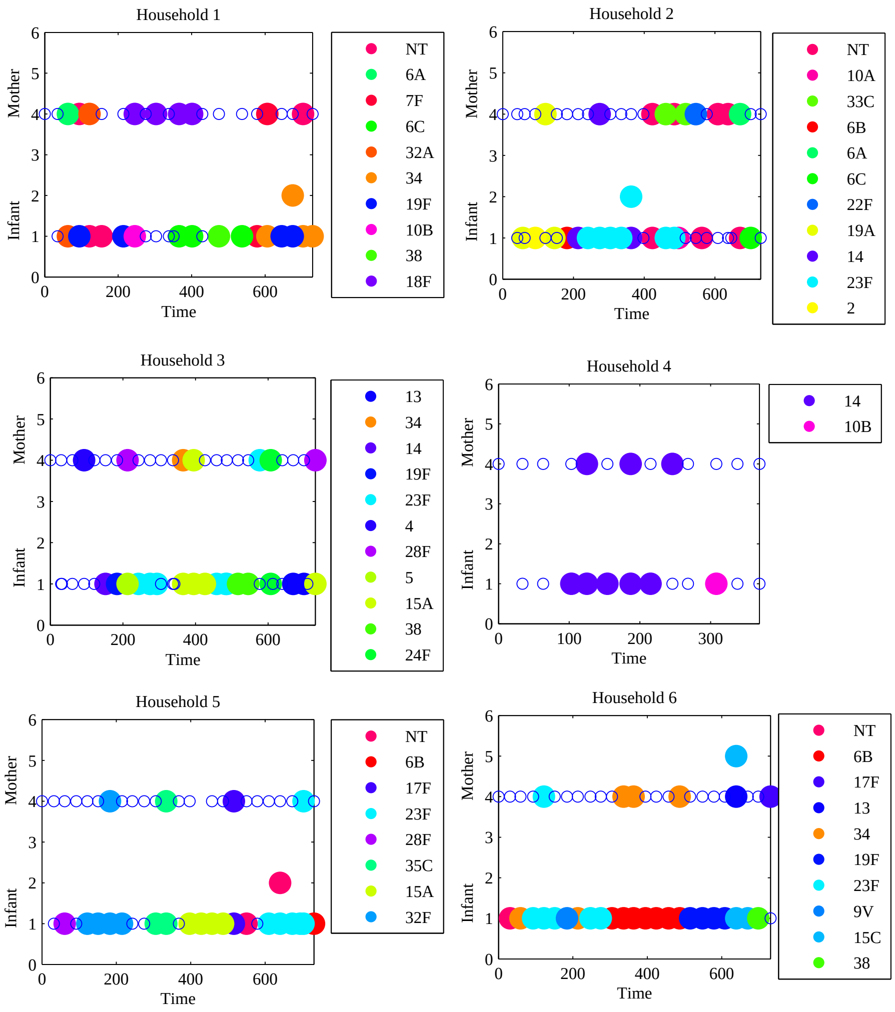

Figure 1: In this figure we show examples of monthly longitudinal observations from mother-infant pairs. The co-colonizing strains, if any, are shown in row 2 for the infant and 5 for the mother.

are observed in a household during the follow-up period, which could indicate that the rate of acquiring new colonizations from outside the household is high. Also, the mother and the infant are often observed to be colonized by same strains, probably due to transmission between the two. Finally, at some occasions, a certain strain is first observed carried by either of the two index individuals, and later observed in the household again, despite not being observed in the index individuals in the meantime. This suggests either a re-introduction of the same strain from the community or, that the strain has been circulating within the household.

2.2 Sequential sampling from the joint posterior distribution

In the methods of approximate Bayesian computation [1], [5], and [6], datasets are simulated with model parameters sampled from the prior, and parameters that yield data similar to the observed data are accepted to the posterior sample. We simulate the model using Gillespie method [7]. When simulating the transmission model described in previous section, we set the first rows of matrices $\boldsymbol{A}$ and $\boldsymbol{I}$ to represent the index individuals in the data. While the states of all the household members are simulated, only the simulation results for the first rows are considered when comparing simulation results to data. As the average household size in Mae La was observed to be 6.4 in [8], we assumed in our transmission model that there were six individuals in total in each of the households. This corresponds to assuming that at each observation time, there were 4 hidden states in the household, corresponding to the unobserved family members. We further assumed that two of the unobserved individuals were infants and two of them were adults, i.e. we set in the model $N_a = 3$ and $N_i = 3$. We denote with $\boldsymbol{D}_h^*(i)$ the hidden state of the household h at the observation time i, that is the joint colonization states of the unobserved individuals, similarly as in equation (1).

Denote the model parameters of the transmission model in equations (1)-(6) jointly with $\boldsymbol{\psi}$. For each considered household h, we perform inference in the joint space of

$$\{\boldsymbol{\psi}, \{\boldsymbol{D}_h^*(i), i = 1, ..., N_h\}\} \tag{2}$$

i.e. for the model parameters and the latent states of the 4 unobserved family members at each observation time of the household h. Inference is performed sequentially, first sampling from $\{\boldsymbol{\psi}, \{\boldsymbol{D}_h^*(i), i = 1\}\}$, then from $\{\boldsymbol{\psi}, \{\boldsymbol{D}_h^*(i), i = 1, 2\}\}$ and so on, until an approximation of the posterior distribution of pairs of the type described in equation (2) is obtained. The sampling is performed by simulating the system states for consequtive observation times, and proposed parameters and latent states are accepted if the transitions in the states of index individuals are similar enough to the transitions in the states of mother and infant in the data. The types of similarity are formally defined as follows:

1. Colonization status of the individual is the same as previously.
2. Previously susceptible individual becomes colonized with a strain that has not yet been observed colonizing either of the index individuals.

3. Previously susceptible individual becomes colonized with a strain that has been observed colonizing certain index individual or individuals t time units ago.

4. Previously colonized individual has cleared the colonization of certain number of colonizing strains.

5. Previously infectious individual becomes co-colonized with a strain that has not yet been observed colonizing either of the index individuals.

6. Previously infectious individual becomes co-colonized with a strain that has been observed in colonizing certain index individual or individuals t time units ago.

A transition in the simulated states of the first rows of $\boldsymbol{I}$ or $\boldsymbol{A}$ are always one of these types. We consider a simulation step to have yielded similar result as in the data, if the transition type is the same. Thus, in effect, the serotype labels are considered exchangeable, and characterized in the fitting only through their previous absence or presence in the household considered. Now the parameters $\boldsymbol{\psi}$ and the consecutive latent states are propagated towards the target distribution (2) as follows:

1. When $i = 1$, sample $\boldsymbol{\psi}$ from prior and $\boldsymbol{D}_h^*(i)$ from initial distribution.

2. Simulate with $\boldsymbol{\psi}$, and with household initial state of the household being $\{\boldsymbol{I}_h(i), \boldsymbol{A}_h(i)\}$ and $\boldsymbol{D}_h^*(i)$ in total $\boldsymbol{t}_h(i+1) - \boldsymbol{t}_h(i)$ time units. If the simulated states of the mother and the infant yield same transitions as in the data, retain the parameter value and the latent states $\{\boldsymbol{D}_h^*(i), \boldsymbol{D}_h^*(i+1)\}$, and collect the $\{\boldsymbol{\psi}, \{\boldsymbol{D}_h^*(i), \boldsymbol{D}_h^*(i+1)\}\}$ into parameter population.

3. Repeat the above steps until a pre-specified sample size is obtained.

4. For each other time interval in the observation times $i = 2, ..., N_h - 1$, repeat, but now sample parameters and hidden states from the population generated in the previous run.

Thus parameters and latent states that have yielded acceptance are sampled for each time interval in the time-series, until the end of the follow-up in for the household at question. Parameters and initial latent states are sampled from the population of accepted parameters and states in the previous time interval. In practice, for each time interval, we run the algorithm until 5000 simulated values are accepted. This approach is analogous to the methods of sequential approximate Bayesian computation [6], since we gradually add more information in the data that is considered, and while adding the information, we use the parameter population (that also contains the latent states of the household members) that was obtained when simulating the previous time interval as the proposal distribution for obtaining the next parameter population.

Finally, the posterior distributions for each separate household together were merged together to obtain a joint posterior distribution for the model parameters.

3 Posterior distributions of the parameters of the within-household-transmission model

3.1 Prior and posterior distributions

As many statistical studies on pneumococcal acquisition dynamics have already been made e.g. [1], [4] and [3], we utilize the existing information and use informative prior distributions on parameters $\theta, \lambda, s_t, s_c$, that correspond to within-host biological processes. Thus we expect the results obtained in the earlier studies to adequately well hold for the host population considered in our study. This is supported by the fact that estimates of these parameters across different studies have also been fairly consistent ([1], [4] [3]). On the other hand, we allocate non-informative prior distributions for the parameters describing infection hazards from household members and from the community, described by β_h and β_c. Obviously, the patterns social interactions and the population density could be very different in societies such as the Nordic countries [3] [1] or UK [4], compared to a society of refugees living in a refugee camp, and thus estimating β_h and β_c is our main interest here.

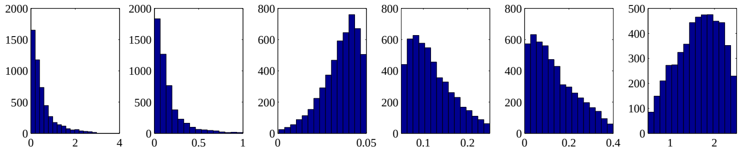

(a) Posterior distribution for model parameters, corresponding to prior distributions defined in Table 3.1

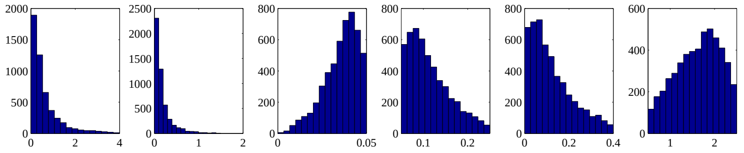

(b) Posterior distribution for model parameters corresponding to a prior distributions in which $\beta_h \sim Uniform(0, 4)$ and $\beta_c \sim Uniform(0, 1.5)$, but other prior distributions are equal to the distributions above.

Figure 2: Posterior distributions for model parameters with the actual prior distribution (upper panels) and with more prior uncertainty for parameters β_c and β_h (lower panels). The panels represent the parameters from left to right in the following order: β_h, β_c, λ, θ, s_t, and s_c.

To ensure that our prior distributions for β_h and β_c actually cover the relevant area of the likelihood, we re-calculated the posteriors with even wider prior distributions for β_h and β_c, which yielded a baseline, or control distribution. In the Table 1 we summarize the prior distributions for the model parameters, summaries of the corresponding posterior distribution and summaries of the control distribution with more prior uncertainty. We have also included references to earlier studies, indicating how the informative prior distributions were chosen. Notice that since informative prior distributions were used, the end points of 95 % CI:s are in many cases close to the limits of the prior distribution. In

	Prior	Ref.	Posterior mean	Control mean	Posterior median	Control median	Posterior 95% -CI	Control 95% -CI
β_h	U(0, 3)	-	0.50	0.61	0.34	0.38	[0.01, 2.14]	[0.02, 2.67]
β_c	U(0, 1)	-	0.15	0.17	0.10	0.11	[0.004, 0.64]	[0.005, 0.77]
λ	U(0, 0.05)	[4], [3]	0.03	0.04	0.04	0.04	[0.008, 0.05]	[0.01, 0.05]
θ	U(0.05, 0.25)	[1]	0.13	0.12	0.11	0.10	[0.06, 0.24]	[0.05, 0.22]
s_t	U(0, 0.4)	[4], [3]	0.16	0.12	0.11	0.10	[0.009, 0.37]	[0.005 , 0.35]
s_c	U(0.5, 2.5)	[4], [3]	1.59	1.61	1.69	1.66	[0.62, 2.42]	[0.64, 2.43]

Table 1: The prior distributions and the summaries of the estimated posterior distributions for the model parameters. Except for parameters β_h and β_c, informative prior distributions were used, and we have chosen the informative prior distributions to be in good agreement with results obtained from previous studies, which are indicated in the 'Reference' column. With control mean and control median we mean the posterior summaries for the same parameters, when the prior distributions for β_h and β_c were the following: $\beta_h \sim Uniform(0, 4)$ and $\beta_c \sim Uniform(0, 1.5)$

Figure **??** we show histograms of the posterior distributions of parameters both for the actual posterior and for the control distribution. By comparison of the histograms and the summaries of the resulting two posterior distributions, it appears that the original prior that was used is not misplaced compared with the support from the data.

3.2 Posterior predictive distributions from the within-household epidemics

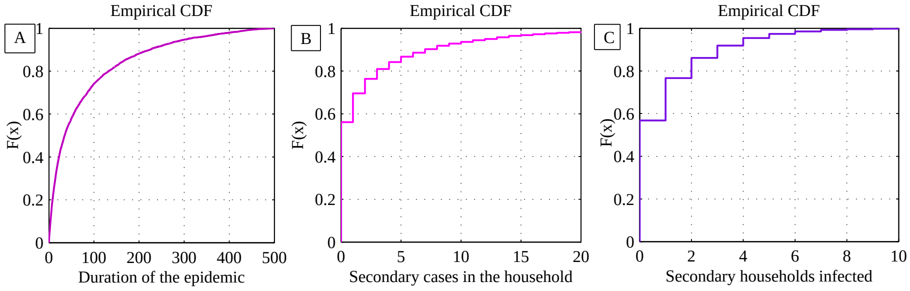

Figure 3: The predicted properties of epidemics of pneumococcus within and between the households. In A we show the prediction for the duration of an epidemic (in days) within a household. In B we show how many additional transmissions occur in the household due to the one infection from outside the household. Finally, in C we show the posterior predictive distribution for how many other households an infected household infects.

The posterior distribution for the within-household dynamics was used to construct a posterior predictive distribution for the length of a within-household epidemic induced by one single infection outside the household. In practice, we sampled model parameters from the posterior and simulated dynamics within

a household first 100 days, and after this we monitored, for how long it takes for within-household epidemics initiated by a single infection to end. This was repeated for independent simulations and the corresponding times were retained in the posterior distribution of the lengths of the epidemics.

When simulating the actual household-to-household transmission trees, we used the above mentioned distribution for sampling lengths of the branches. We made the assumption of endemic equilibrium, in which the infected households were expected to infect on average one other household. This, together with the assumption of households being equally infectious regardless of the number of household members that are infectious, allowed us to solve the rate at which new branches are born in the branching process models. In Figure 3, we summarize the posterior predictive distributions obtained, describing the characteristics of the transmission process and the transmission trees.

3.3 Endemicity and possible seasonality of pneumococcal infections

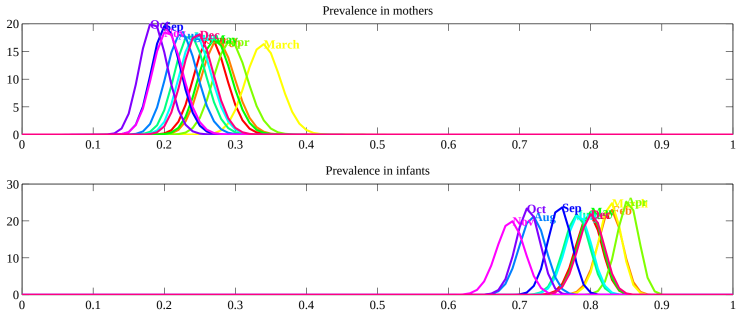

Figure 4: The posterior distribution of the parameters describing for each month the probability of being colonized with pneumococcus for mothers and infants.

Having a uniform rate at which households infect each other implies that there is no seasonality in transmissions. This could yield seriously biased results, if in reality there would be seasons during which transmissions would be more frequent. One possible source of seasonality for the data that originates from South-East Asia, could be the weather, that alternates between cool, hot and rainy seasons. To check whether such an assumption would hold, we did a simple inference on how the calendar month affects the probability that a person is colonized. For each month t, we assumed that the probability that a person was colonized was described by p_t^m for mothers and p_t^i for infants. For each of the 2×12 parameters we gave a $Beta(1, 1)$ prior distribution, that corresponds to uniform distribution. Posterior distribution for parameter p_t^i is then a Beta distribution with hyperparameters $1 + n_t^i(c)$ and $1 + n_t^i(c) + n_t^i(e)$, where $n_t^i(c)$ corresponds to the number of times an infant at month t was observed colonized and $n_t^i(e)$ corresponds to the number of times an infant at month t was observed not colonized. In Figure 4 we show the resulting posterior distributions. From the figure we see that while there are small differences in prevalence troughout the year, colonization being most prevalent in spring and least prevalent in

the autumn both in mothers and infants, no dramatic differences do exist. In addition, high prevalence does not directly indicate high transmission. This is because the pneumococcal strains compete with each other. Therefore, in the months of high prevalence while there is more possible donors of infection, it is less easy to succefully colonize a host. In summary, the data do not suggest presence of a non-negligible seasonal component that should be taken into account in the transmission modeling.

4 Classification of plausible transmission pairs and partitioning of the data

4.1 Plausible transmission pairs

Since there were no confirmed cases of transmission, we adopted a fairly parsimonious assumption and assumed that if the same serotype was observed in infant and mother living in the same household, there was a transmission between the two. This is based on the prior knowledge that the serotypic strain remains unchanged in transmission, and that the transmissions are frequent between infant and mother. Such pairs of observations, can be considered as training data of considering transmission pairs. To the actual training data, we retain the number of differences in the single nucleotide polymorphic sites (SNPs) and the time between the observations. We obtain more training examples by collecting all the consecutive observations of same serotype persisting to colonize same host. A scatterplot of such training examples is shown in Figure 5. As seen from the figure, there is not a very clear temporal signal in the accumulation of differences in genomes in transmission pairs, nor between the observations collected from the same host. We hypothesize that this is explained by relatively large within-host population sizes and not very dramatic transmission bottlenecks, that would reduce this population size at the time of transmission.

It is reasonable to assume that observations potentially due to transmission between *different households* should be similar as in the training data in terms of their genetic variability. For classifying observations as similar we fit a linear model for genotypic differences as a function of time to the training data. We then classify a transmission to be *plausible* between a pair of isolates in different households, if the genotypic differences between two isolates was less or equal than predicted by the linear model. This results in classifier that tells for each pair of observations in different households, whether it is plausible that the observations could be due to transmission. The classifier does not play a very important role here, and in principle we could even classify every pair of observations as plausible transmissions, and then infer using our framework the actual posterior probability of transmission between them. However, as explained in the next section, to obtain smallest possible clusters of data to be analysed separately, which simplifies the computational challenges, we aim at only considering putative pairs of transmission which could be expected to have non-negligible posterior probability of being due to direct transmission.

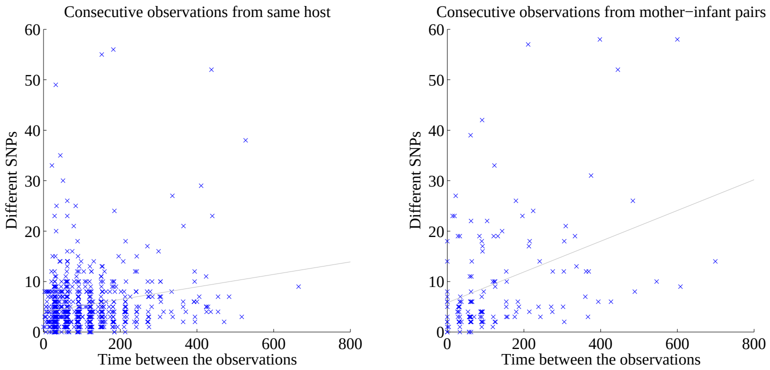

Figure 5: The genotypic distances between two different observations that were both same serotype. In the first panel pairs of observations collected from the same host and in the second panel from mother-infant pairs in the same household.

4.2 Clustering the data

When defining the branching process model for transmission trees in Section 2.2 of the main article, we assumed that each infectious household had a constant rate of infecting another household in the population. Therefore, each branch of the complete branching process is giving birth and dying out independently of the other branches. Such an assumption for the transmission model implies that the data can also be clustered into subsets, over which the likelihood of the complete data factorizes. This clustering is induced by the underlying transmission tree. Namely, if the partitions are such that isolates in distinct clusters originate from distinct subtrees of the transmission tree, then the factorization property holds. With distinct subtrees we mean that no other isolates than the isolates in the particular cluster are descending along the transmission tree from the host that is the transmission-MRCA for the isolates in the cluster. Therefore, when considering the posterior distribution of transmission trees initiated by the transmission-MRCA of a particular cluster, isolates in the other clusters yield no likelihood contribution. Obviously, there may exist many possible data partitions having this property. The most detailed such partition consists of having each isolate in its own cluster, and the coarsest partition clusters all isolates within a single cluster. The isolates within clusters can be analysed independently observations in the other clusters, using the approach described in previous sections. Since for a smaller data set the probability of generating a transmission tree with positive weight will be larger, the purpose of the clustering here was to identify smallest possible sets of data for the analysis. Thus, we cluster the data into the finest possible clustering in which the isolates among which transmission is plausible are clustered in same cluster, and which still satisfies the above criteria. Clearly, as the transmission tree in general is unknown, the correct clustering will be unknown. We will adopt a heuristic approach for the clustering, which we expect to result in similar results as the one described above. For this, we utilize the training data for transmission pairs, described in above, and the parsimony tree of the genotypic isolates.

In detail, we cluster the data as follows. First we identify all the pairs of

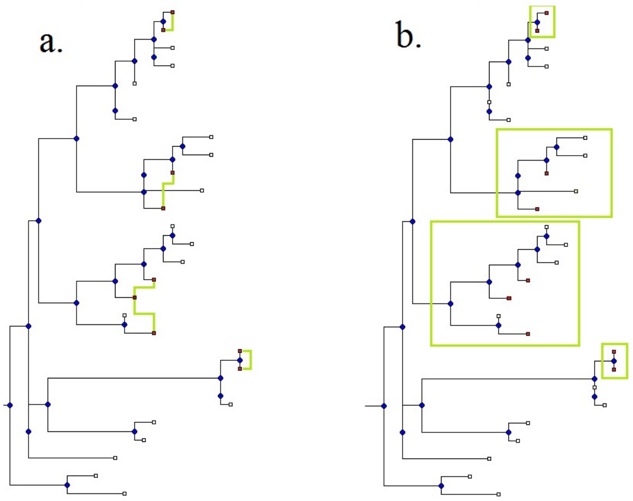

Figure 6: A schematic illustration about how the data can be partitioned into transmission clusters (explained in detail in the text). The leaf nodes, represented by squares at the tips of the phylogenetic tree, correspond to observed isolates. Isolate pairs, between whom transmission was considered plausible are connected with a green line (panel a). In panel b we show the corresponding transmission clusters with green rectangles. The isolates within a tranmission cluster were analyzed as independent data sets.

isolates in different households, between whom transmission pathway is possible according to the classification criteria described in the main article in Section 2.1. This defines a (nondirected) network of possible transmissions between the households in the data. Then, we cluster together all the connected components in this network into clusters of their own, which we call *pre-clusters*. Finally, for each pre-cluster we potentially add further isolates based on the parsimony phylogenetic tree constructed from genotypic isolates. In particular, for each pre-cluster, we take the minimal subtree of the complete phylogenetic tree that contains all the isolates of the pre-cluster and define the actual cluster to contain all the leaves of this tree. We illustrate the idea of the clustering in Figure 4.2. This is based on the assumption that the host carrying the MRCA of certain isolates is the ancestor along the transmission tree to the hosts carrying isolates in the data cluster, and is not ancestor with respect to the transmission tree to any other isolates in the data.

This data partitioning results in each isolate being assigned to a unique cluster. Some clusters might only contain one isolate, indicating that no plausible transmission pathway can be identified between them and other isolates in the data. Conversely, if two or more isolates are clustered together, then each such cluster contains at least one pair of isolates between whom a direct transmission or a direct descendancy along the transmission tree would be possible. In the actual analysis of the clusters, the sampling scheme described in Section 2.4. in the main article is used. For the genealogies, we only consider the posterior

distribution of TMRCA for the isolates in the given cluster and the importance weights are then calculated accordingly. Simulations of transmission trees are initiated for each cluster with one branch alive at the earliest time of the sampled TMRCA. The transmission tree is simulated, conditional on non-extinction, until the last observation time t_e present in the data.

From some of the households several isolates of same strain were collected. As in our observation model we assumed only single observation from each household, we merged all the isolates from a single household into one observation. This was done by defining the observation time for a household to be the first occasion a certain clone was detected in it, for those households from which several observations of the same strain were available. In such cases the divergence times of genotypic lineages in different households, described by $\boldsymbol{T}_{\boldsymbol{D}^g}$, were inferred conditional on the average genotypic differences between observations from the households.

5 Alternative models for observations

As defined in the main article, if k isolates were observed in the data, then the importance weight of a proposed tree Z can be calculated by summing the likelihood of observations over all the k-sets of the tree. However, only such combinations $\boldsymbol{x} \in S_Z(k)$ that are consistent with the data can yield positive contribution to the importance weight. We define a set of branches $\boldsymbol{x}$ to be *consistent with observations*, if:

1. for all $i \in 1, ...k$ the corresponding branch $\boldsymbol{x}(i)$ is alive at the observation time t_i.
2. for all pairs of branches $i, j \in \boldsymbol{x}$ it holds that $\boldsymbol{T}_Z(\boldsymbol{x}(i), \boldsymbol{x}(j)) \geq \boldsymbol{T}_{x(i),x(j)}$.

Above $\boldsymbol{T}_Z(\boldsymbol{x}(i), \boldsymbol{x}(j))$ denotes for the time at which lineages of branches $\boldsymbol{x}(i), \boldsymbol{x}(j)$ coalesce in the transmission tree. We introduce an indicator function $\mathbb{1}_{\{\boldsymbol{t},\boldsymbol{T}\}}(\boldsymbol{x}, Z)$ in the space of $\boldsymbol{x} \times Z \to \{0, 1\}$ that takes value one if a set of branches is consistent with data, and zero otherwise. In the following subsections we define how each consistent set of branches $\boldsymbol{x}$ is weighted under the alternative observation models.

5.1 Model 1

In Model 1 infectious periods are observed with probability p, independently of each other. Therefore the probability of observing k branches from a tree Z, which has n extant branches during the follow-up equals:

$$P(\{k = O(Z)\}) = Binom(n, k, p) \tag{3}$$

In Model 1 we assume that the actual time that a branch was observed, is uniformly distributed over the time the branch was extant. For any Z, there is a corresponding set of branch lengths $\boldsymbol{l}$, in which for any branch i of Z, the branch length is given by $\boldsymbol{l}(i)$. Using this notation, the conditional probability of observing specific branches $\boldsymbol{x}$ at specific times $\boldsymbol{t}$ from Z equals:

$$P(\{\boldsymbol{x}, \boldsymbol{t}|Z\}|k = O(Z)) = \frac{1}{|S_Z(k)|}\left(\mathbb{1}_{\{\boldsymbol{t},\boldsymbol{T}\}}(\boldsymbol{x}, Z)\prod_{i=1}^{k}\frac{1}{\boldsymbol{l}(\boldsymbol{x}(i))}\right) \tag{4}$$

The term $1/|S_Z(k)|$ in the above expression corresponds to the probability that a certain k-set of branches was observed. This was under the assumed observation model equal for every set, and thus equals inverse of the number of different k-combinations of branches from Z. The indicator function ensures that the corresponding branches of the k-set were alive at the observation times. Lastly, the product evaluates the probability that the infections were observed at the specific observation times, which equal to the inverse of the length of the branch, since each observation time was assumed to be uniformly distributed along the branch.

5.2 Model 2

In Model 2 branches are also assumed to be observed with probability p, and thus equation (3) holds. However, individual combinations of branches are weighted differently in $P(\{\boldsymbol{x}, \boldsymbol{t}|Z\}|k = O(Z))$, compared with Model 1. Let $\boldsymbol{t}_s(i)$ denote for the time when the branch i was born (i.e. the infection was initiated), and let $\boldsymbol{t}_e(i)$ denote for the time it ended (the infection ended). In addition, denote with $g(x; \alpha, \beta)$ the probability distribution function of a gamma distribution with shape α and scale β, and with $F(x; \alpha, \beta)$ the corresponding cumulative distribution function. Finally, denote with $\tau(\boldsymbol{t}(i)|\boldsymbol{x}(i))$ the probability of branch i to be observed at time $\boldsymbol{t}(i)$. We define $\tau(\boldsymbol{t}(i)|\boldsymbol{x}(i))$ to be the following under Model 2:

$$\tau(\boldsymbol{t}(i)|i) = \begin{cases} \dfrac{1}{t_e(i) - t_s(i)}, & \text{if} \quad t_e(i) - t_s(i) < 30 \\ \dfrac{g(t(i) - t_s(i); 3, 10)}{F(t_e(i) - t_s(i); 3, 10)}, & \text{if} \quad t_e(i) - t_s(i) > 30 \end{cases} \tag{5}$$

The probability density corresponding to the case $t_e(i) - t_s(i) > 30$ corresponds gamma distribution that is truncated on the interval $[0, t_e(i) - t_s(i)]$, the lifetime of the branch at question. From (5) it follows:

$$P(\{\boldsymbol{x}, \boldsymbol{t}|Z\}|k = O(Z)) = \frac{1}{|S_Z(k)|} \mathbb{1}_{\{\boldsymbol{t}, \boldsymbol{T}\}}(\boldsymbol{x}, Z) \prod_{i=1}^{k} \tau(\boldsymbol{t}(i)|\boldsymbol{x}(i))$$

5.3 Model 3

Under Model 3, we assume that branches are observed with probability that is proportional to their length. To make the conclusions comparable to the other models, we defined the probability that a branch i was observed to be $c \times l(i)$, where we set c so that on average p branches get observed. This was possible as we had a distribution for the branch lengths $\boldsymbol{l}$. Thus, since each branch had a probability proportional to its length to be observed and the actual observation time of the infection was uniformly distributed over the length of the branch, we obtain:

$$\begin{aligned} P(\{\boldsymbol{x}, \boldsymbol{t}|Z\}|k = O(Z)) &= \frac{1}{|S_Z(k)|} c^k \times \mathbb{1}_{\{\boldsymbol{t}, \boldsymbol{T}\}}(\boldsymbol{x}, Z) \times \prod_{i \in \boldsymbol{x}} \boldsymbol{l}(\boldsymbol{x}(i)) \prod_{i \in \boldsymbol{x}} \frac{1}{\boldsymbol{l}(\boldsymbol{x}(i))} \\ &= \frac{c^k}{|S_Z(k)|} \times \mathbb{1}(\boldsymbol{x}, Z) \end{aligned}$$

Here each consistent *set of branches* in a given tree acquires the same weight. However, the probability of observing k branches from a tree Z depends now on the particular branch lengths:

$$P(\{k = O(Z)\}) = \sum_{\boldsymbol{x} \in S_Z(k)} \prod_{i \in \boldsymbol{x}} (c \times \boldsymbol{l}(\boldsymbol{x}(i))) \prod_{j \notin \boldsymbol{x}} (1 - c \times \boldsymbol{l}(j)$$

Thus the probability of observing k branches from a tree Z is obtained by summing over all the possible k-sets of branches of the tree the probability of observing the particular set and not observing any other branch.

5.4 Model 4

In this model we assume the data collection process is similar as in the Model 1. However, we introduce a different model for the coalescence of lineages within a transmission tree $P(\{\boldsymbol{T}|\boldsymbol{x}, \boldsymbol{t}, Z\}$. In particular, for every consistent $\boldsymbol{x}$, and every pair of branches $i, j \in \boldsymbol{x}$, there exists most recent transmission common ancestor in Z that is the most recent host in which the transmission lineages of i and j coalesce.

Let us denote with $\chi_Z(\boldsymbol{T}_{i,j}, i, j)$ a function that calculates for any pair of branches of the tree Z the number of transmission events from the transmission most recent common ancestor of i and j, that are required to traverse backwards in time along Z to reach $\boldsymbol{T}_{i,j}$. In Figure 7 we illustrate how the values of χ_Z are calculated. In essence, χ_Z counts the number of hosts genotypic lineages of i and j *both* traverse through until coalescence, minus one. Subsequently, we define

$$P(\{\boldsymbol{T}|\boldsymbol{x}, \boldsymbol{t}, Z\}) = q^N, \tag{6}$$

where:

$$N = \sum_{i,j \in \boldsymbol{x}} \chi_Z(\boldsymbol{T}_{i,j}, i, j)$$

If transmission bottlenecks are known to be severe, q is to be set close to zero, while if many isolates are transmitted in a transmission event, q is to be set close to one. In our application, we used $q = 0.7$, that was chosen since the within-host diversity of pathogens was observed to be high, as seen in Figure 5. Unfortunately the actual value of q is unknown and would require a detailed study on this matter.

5.5 Model 5

In model 5 the data are collected as in model 3 and coalescence is modelled as in Model 4.

5.6 Model 6

In model 6 the data are collected as in model 2 and coalescence is modelled as in Model 4.

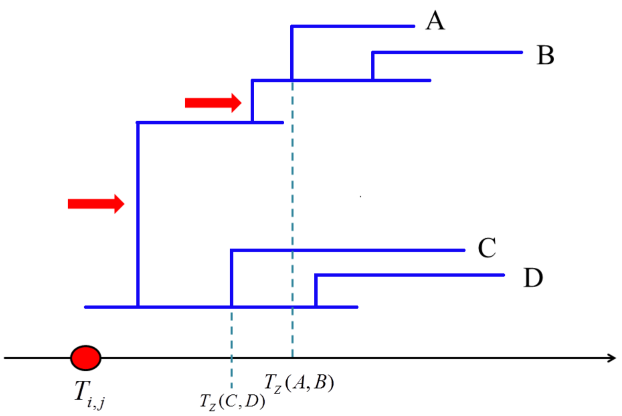

Figure 7: Above we illustrate how the number of transmission bottleneck events is calculated from a given transmission tree and a given time to most recent common ancestor for two isolates T_{ij}. Above both pairs (A,B) and (C,D) are consistent with T_{ij}, since both $T_Z(A,B)$ and $T_Z(C,D)$ are larger than T_{ij}. On the other hand, the lineages of A and B have to traverse together in two transmission events in order to coalesce as early as T_{ij}, while the lineages of C and D do not have to survive any transmission bottleneck events to do so.

6 Sampling the coalescence times of the genotypic lineages

6.1 Construction of phylogenetic trees without recombination

Our goal was to use information on single nucleotide polymorphisms (SNPs) for inferring transmissions. Two types of events create SNPs, mutation, that introduces one change in the genome at a time, and recombination, in which several SNPs can be introduced into the genome simultaneously. We used the method described in [9] to separate SNPs from the two sources, and only the SNPs that were expected to be created by mutation were used in our analysis. Furthermore, using the method we constructed a maximum likelihood tree with parsimonious SNPs reconstruction. This yields a phylogenetic tree for the isolates, in which the branch lengths can be interpreted as accumulation of new SNPs.

6.2 Sampling the unknown coalescence times of the phylogenetic tree

We assumed that the branching order in each constructed tree was the true order in the actual unknown genealogy of the isolates. However, we wanted to estimate the posterior distribution of the times of the ancestral nodes of the phylogenetic tree. For this, we constructed a Markov chain that samples from the posterior distribution of the times of nodes.

We denote with T a given tree with n leaves, and the unknown times of the inner nodes $\boldsymbol{t} = (t_{n+1}, ..., t_{2n-1})$, where t_{2n-1} is the timing of the root. Assume that the leaves have been observed at $t_1, ..., t_n$. We can calculate the likelihood of $\boldsymbol{t}$ and T give the lengths of the parsimony tree D as a product:

$$p(D|T, \boldsymbol{t}) = \prod_{i=1}^{2n-2} p(m_i | t_i - t_{pa(i)}).$$

Where $pa(i)$ is the parent of node i in tree T, m_i the number of new SNPs occurring between $pa(i)$ and i in T and $p(m|t_i - t_{pa(i)})$ is the probability density function of the Poisson distribution at m_i with the parameter $dt \times u$, where u is the mutation rate. In our application here, we used a fixed mutation rate of 1.57×10^{-6} per site per year, that was reported for pneumococcus in an earlier study [9].

Now, having a likelihood for the tree and the times of inner nodes, we propose a Metropolis-within-Gibbs type of MCMC-algorithm [10]. At each step, it samples a random inner node of the tree, and given the current times of the other inner nodes of the tree, the algorithm proposes a new time for the sampled node. Proposal is accepted according to an Metropolis-Hastings transition kernel:

1. Set $j = 1$, set t_{min}, that is the minimal time for the coalescence times of all lineages in the data and set the desired sample size N

2. Sample a random inner node i of the tree.

3. If the node i is the root node, sample a time $\boldsymbol{t}^*(i)$ for the node uniformly from $(t_{min}, min\{\boldsymbol{t}(c_1(i)), \boldsymbol{t}(c_2(i))\}$, where $c_1(i)$ and $t(c_2(i)$ are the current times of the child nodes of the node i. Else if the sampled node is not the root, then sample the $\boldsymbol{t}^*(i)$ uniformly from interval $[\boldsymbol{t}(pa(i)), min\{\boldsymbol{t}(c_1(i)), \boldsymbol{t}(c_2(i))\}]$, where $\boldsymbol{t}(pa(i))$ is the current time of the parent node of the node i.

4. Calculate the Metropolis-Hastings-ratio $m_{\boldsymbol{t},\boldsymbol{t}^*} = \frac{p(D|T,\boldsymbol{t})}{p(D|T,\boldsymbol{t}^*)}$ and accept $\boldsymbol{t}^*$ with probability equal to $m_{\boldsymbol{t},\boldsymbol{t}^*}$ and retain $\boldsymbol{t}^*$ to the posterior and set $\boldsymbol{t} = \boldsymbol{t}^*$. If $\boldsymbol{t}^*$ is not accepted, retain $\boldsymbol{t}$ to the posterior.

5. Repeat 2.-4. until sample in total N instances of $\boldsymbol{t}$ are included in the posterior sample.

Notice that above, when Metropolis-Hastings ratio is calculated, proposal distributions and prior distributions cancel out, if uniform distributions are used for both of them. The output of the algorithm is a sample from the posterior distribution of $\boldsymbol{t}$.

7 Geographic spread of infections

To illustrate the effect of an observation model, we estimated the posterior distribution for transmission links between the pairs of observations from different households conditional on each of the six observation models. The distribution for the links appears different in light of the different models. Under each model, we ranked the pairs of households according to the average number of

transmission links between them. Then, by investigating the first ranked pairs under-each model, and the geographic distance between them, we noticed that while different pairs of households seem most plausible for direct transmission, still under each of the models the best ranked pairs are also in close proximity to each other. In Figure 8 we show for the 6 different models the 15 top-ranking pairs of households in the map. Above each figure, we also show the mean distance between them. In the last panel of Figure 8 we show the distribution for mean distance for 15 pairs of randomly chosen households. These observations suggest that the pairs of households that are closely related with respect to transmission links are also more closely related in terms of their geographical distance. A random sample of 15 pairs of households would extremely rarely have a mean distance of ~ 300 meters between the households, which was the predicted mean distance for most of the observation models. To show that this observation is not an artefact depending on choosing the 15 top-ranked pairs, and not for instance 30 top-ranking pairs, we show in Figure 9 how the mean depends on the number of top-ranked pairs from which the mean is calculated.

Finally, we show in figure 10 the 15 top-ranking pairs of transmissions, when the model average for the transmission links was considered as the ranking criteria. We also show the corresponding number of transmission links, and the distance between the corresponding households.

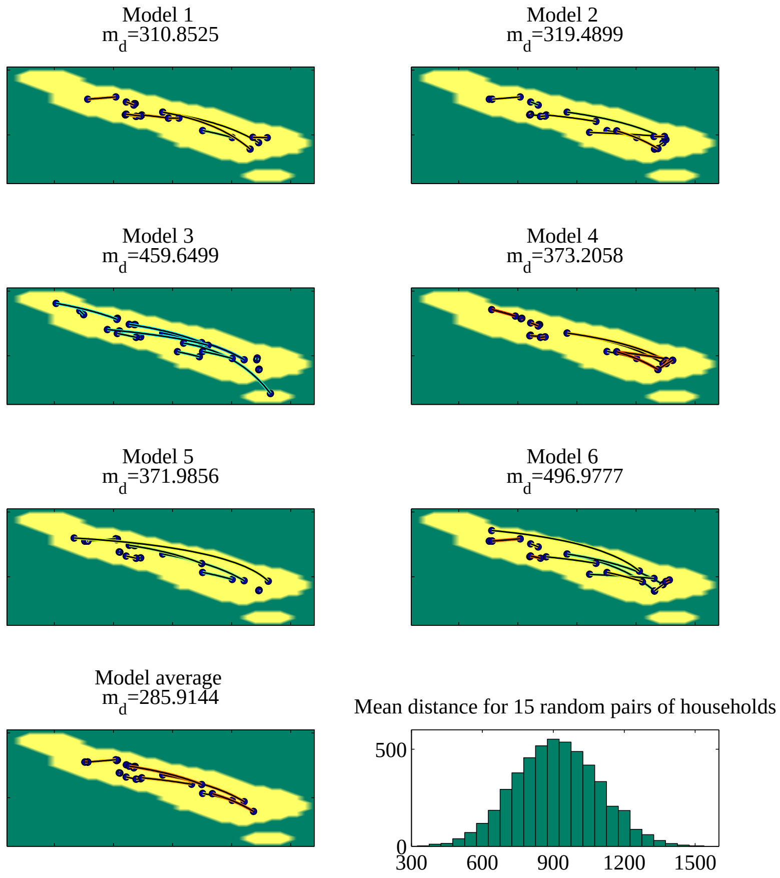

Figure 8: The pairs of households, between whom the predicted number of transmission events was among the 15 top-ranked. The yellow area corresponds to the populated area in the refugee camp, and the points correspond to households, connected by lines presenting possible transmission. Above each figure we show the mean distance of of the shown pairs of households.

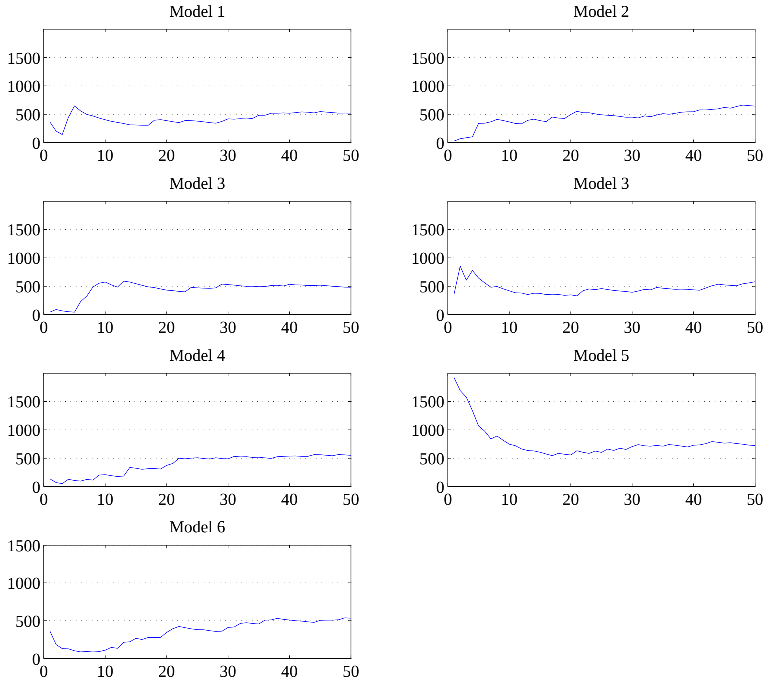

Figure 9: For each of the observation models, we show how the mean distance between the first ranked pairs of transmission depends on how many of the top-ranked pairs are considered.

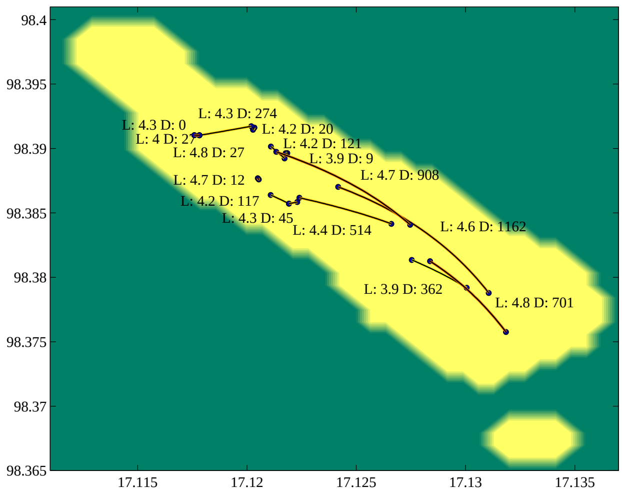

Figure 10: The pairs of households, between whom the model average of the predicted number of transmission events was below the 20 %-quantile. The yellow area corresponds to the populated area in the refugee camp, and the points correspond to households, connected by lines presenting possible transmission. Close to each line we show the number posterior mean for the predicted transmission links (L), together with the distance between the corresponding households in meters (D). The pairs closest to each other have a visually negligible distance in the figure.

8 Supplementary figures

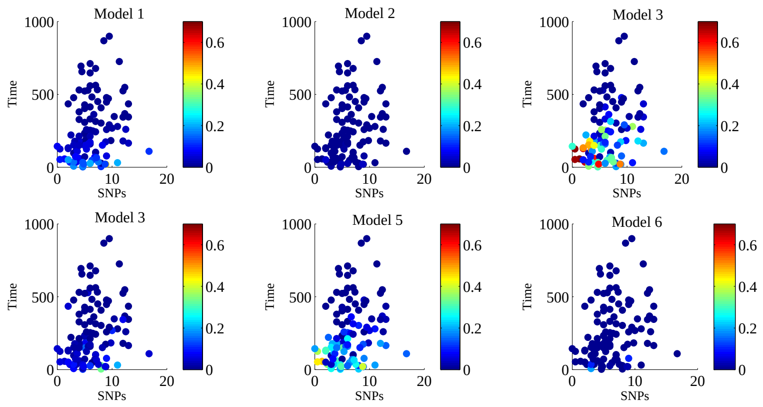

Figure 11: The posterior probability of direct transmission for pairs of observations in the data for whom the transmission was considered plausible under the six different models for observations.

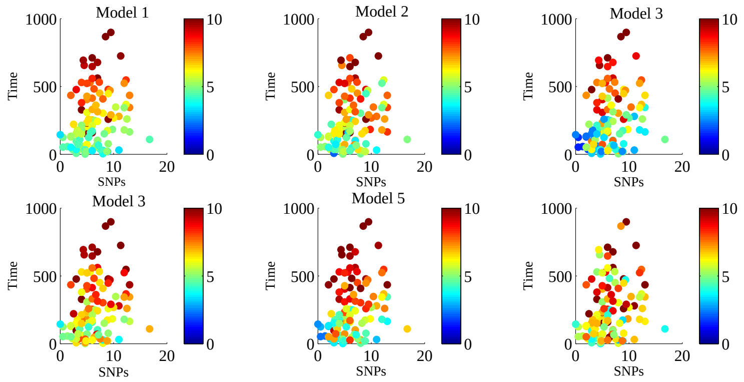

Figure 12: The posterior mean for the number of transmission links between the pairs of observations in the data for whom the transmission was considered plausible under the six different models for observations.

References

[1] Numminen E, Cheng L, Gyllenberg M, Corander J. 2013 Estimating the transmission dynamics of Streptococcus pneumoniae from strain prevalence data *Biometrics* (DOI: 10.1111/biom.12040)

[2] Regev-Yochay G, Raz M, Dagan R, Porat N, Shainberg B, Pinco E, Keller N, and Rubinstein E. 2004 Carriage of Streptococcus pneumoniae by Adults and Children in Community and Family Settings *Clinical Infectious Diseases* **38**, 632-639. (doi: 10.1086/381547)

[3] Auranen A, Arjas E, Leino T, Takala AK. 2000 Transmission of Pneumococcal Carriage in Families: A Latent Markov Process Model for Binary Longitudinal Data. *Journal of the American Statistical Association* **95**, 611-636. (doi:10.1214/08-AOAS230)

[4] Melegaro A, Choi Y, Pebody R, Gay N. 2004 Pneumococcal Carriage in United Kingdom Families: Estimating Serotype-specific Transmission Parameters from Longitudinal Data. *American Journal of Epidemiology* **166**, 228-235. (DOI:10.1017/S0950268804001980)

[5] Beaumont, MA. 2010 Approximate Bayesian Computation in Evolution and Ecology. *Annual Review of Ecology, Evolution, and Systematics* **41**, 379-406. (doi: 10.1146/annurev-ecolsys-102209-144621)

[6] Sisson S, Fan Y, Tanaka M. 2007 Sequential Monte Carlo without likelihoods. *Proceedings of the National Academy of the Sciences* **104**, 1760-1765. (doi: 10.1073/pnas.0607208104)

[7] Gillespie, DT. 1976 A General Method for Numerically Simulating the Stochastic Time Evolution of Coupled Chemical Reactions. *Journal of Computational Physics* **22**, 403-434. (doi:10.1016/0021-9991(76)90041-3).

[8] Banjong O, Menefee A, Sranacharoenpong K, Chittchang U, Eg-kantrong P, Boonpraderm A, Tamachotipong. 2003 Dietary assessment of refugees living in camps: A case study of Mae La Camp, Thailand *Food and Nutrition Bulletin* **24**, The United Nations University

[9] Croucher NJ et al. 2011 Rapid pneumococcal evolution in response to clinical interventions. *Science* **331**, 430-434. (doi:10.1126/science.1198545)

[10] Tierney L. 1994 Markov chains for exploring posterior distributions. *The Annals of Statistics.* **22**, 1701-1728
